# Effective treatment of TNFα inhibitors in Chinese patients with Blau syndrome

**DOI:** 10.1186/s13075-019-2017-5

**Published:** 2019-11-12

**Authors:** Jing Chen, Yi Luo, Mengzhu Zhao, Di Wu, Yunjiao Yang, Wen Zhang, Min Shen

**Affiliations:** 1 Department of Rheumatology, Peking Union Medical College Hospital, Chinese Academy of Medical Sciences & Peking Union Medical College, Key Laboratory of Rheumatology and Clinical Immunology, Ministry of Education, Beijing, 100730 China; 2 Present Address: Department of Rheumatology, Chongqing Three Gorges Central Hospital, Chongqing, 404000 China; 3 Department of Rheumatology, Peking Union Medical College Hospital, No.1 Shuaifuyuan, Dongcheng District, Beijing, 100730 China

**Keywords:** Systemic autoinflammatory diseases, Nucleotide-binding oligomerization domain, Blau syndrome, TNFα inhibitors

## Abstract

**Objectives:**

Blau syndrome (BS) is a rare dominantly inherited autoinflammatory disorder associated with mutations in the nucleotide-binding oligomerization domain containing 2 (*NOD2*) gene. Biologic therapy of BS yielded diverse results. We aimed to evaluate clinical features and outcomes of Chinese patients with BS who were treated with tumor necrosis factor (TNF)α inhibitors.

**Methods:**

A total of four patients with BS were diagnosed and treated with infliximab (IFX) at the Peking Union Medical College Hospital during 2015 to 2018 and were followed up for 18 months. All patients were systematically studied for treatment outcomes including the clinical manifestations and inflammatory markers. We also conducted a comprehensive literature review about TNFα inhibitor therapy in BS.

**Results:**

Four BS patients were all Chinese Han, and three were women. The mean age of disease onset was 4 ± 3.5 years, and the mean time of diagnosis delay was 19 ± 11 years. All patients received IFX plus methotrexate, and all achieved clinical remission of skin lesions and polyarthritis rapidly, as well as normalization of erythrocyte sedimentation rate and C-reactive protein and improvements in inflammatory cytokines, patient visual analogue scale, physician global assessment, and Short Form (SF)-36, at the first follow-up of 6 months. The disease relapsed in two patients after they lengthened the interval of IFX and discontinued methotrexate. According to the 38 English-language publications, 62 patients with BS were reported who underwent TNFα inhibitor therapy, including IFX used in 31, adalimumab in 24, and etanercept in 7. IFX was well tolerated in 27 patients, while 2 still had uveitis, and the other 2 experienced an adverse drug reaction.

**Conclusions:**

Early recognition and effective treatment of BS are very important to avoid irreversible organ damage. TNFα inhibitors such as IFX may be a promising approach for BS patients who have unsatisfactory response to corticosteroids and traditional disease-modifying antirheumatic drugs.

## Introduction

Blau syndrome (BS; OMIM 186580) is a rare dominantly inherited autoinflammatory disorder [1], associated with mutations in the nucleotide-binding oligomerization domain containing 2 (*NOD2*) gene [2]. The main features of BS include the clinical triad of granulomatous dermatitis, arthritis, and recurrent uveitis with childhood onset [3]. BS is mainly seen in Caucasian patients [4, 5], but has also been sparsely reported in the Chinese population [6, 7]. We have documented the two by far the largest Chinese pedigrees with 13 members affected [8]. The heterozygous R334W variant in the *NOD2* gene was identified in both families. The considerable percentage of patients who had only one component of the classical triad further complicates the diagnosis of BS in clinical practice.

It is critical to control the eye and joint involvements to improve the prognosis of BS. The use of non-steroid anti-inflammatory drugs (NSAIDs), corticosteroids, and in refractory cases, immunosuppressive agents, such as methotrexate and azathioprine, has been reported to date. Recently, biologic agents such as interleukin (IL)-1 blockers and tumor necrosis factor (TNF)α inhibitors have demonstrated promising effects in some cases of unsatisfactory response [9, 10]. We have reported a 13-year-old Chinese boy with refractory BS who maintained an effective response to tocilizumab [7]. After a systematic literature review, we found that biologic therapy of BS yielded diverse results [7], which may be due to the different genotypes and phenotypes of BS. Meanwhile, the small number of patients in those studies may also make it difficult to give a definite conclusion.

In this study, we describe four Chinese patients with BS who were treated with infliximab (IFX) during 2015 to 2018, in Peking Union Medical College Hospital, and also reviewed the published English literature of TNFα inhibitor therapy in this disease.

## Patients and methods

All these four Chinese BS patients were referred to and followed up for 18 months in our tertiary medical center, including three patients we have reported before [8]. Complete medical records and detailed data were collected and documented. Due to unavailability of IL-1 antagonist therapies in China, they were treated with IFX. We assessed the response to therapy by monitoring inflammatory markers, which include white blood cell count (WBC), C-reactive protein (CRP), erythrocyte sedimentation rate (ESR), and TNFα, IL-1β, and IL-6 levels, and observing clinical manifestations by patient visual analogue scale (VAS), physician global assessment (PGA), and Short Form (SF)-36. We performed a systematic literature search in PubMed using the terms as “Blau syndrome” OR “autoinflammatory disease AND TNFα inhibitors” OR “Blau syndrome AND TNFα inhibitors” OR “Blau syndrome AND infliximab.” Totally, there were 249 articles published in PubMed ranging from September 1991 to March 2019, of which 203 articles were excluded for not reporting TNFα inhibitors used in BS. Among the remaining 46 articles, the full texts of 8 articles were unavailable. Ultimately, 38 articles containing case reports of BS patients receiving the treatment modalities were reviewed.

This research was approved by the Institutional Review Board of Peking Union Medical College Hospital and performed according to the Declaration of Helsinki. Informed consents were obtained from all participants. Whole exome sequencing by next-generation sequencing was performed in the Center for Genetic Testing, Joy Orient Translational Medicine Research Centre Co., Ltd., Beijing, China.

## Results

The demographic data, clinical phenotypes, and laboratory features of these four patients were summarized in Table 1. The mean age of disease onset was 4 ± 3.5 years. The mean age at diagnosis was 22 ± 14 years, and the mean time of diagnosis delay was 19 ± 11 years.

**Table 1:** Demographic and clinical features of four Chinese patients with BS

Patients	1	2	3	4
Gender	Female	Male	Female	Female
Age at diagnosis (years)	32	8	36	25
Age at onset (years)	6	0.5	7	6
Ethnicity	Han	Han	Han	Han
Family history	+	+	+	–
Clinical features				
Joint	+	+	+	+
Skin	+	+	+	–
Eye	+	–	+	+
Fever	+	–	–	–
*NOD2* variants	R334W	R334W	R334W	R334Q
Laboratory findings				
WBC (× 10^9^/L)	4.15	9.5	16.3	7.99
CRP (mg/L)	3.48	20.0	33.01	3.4
ESR (mm/h)	16	5	48	11
IL-1β (pg/ml)	78.0	77.8	70.2	108.5
TNFα (pg/ml)	114.0	245.0	156.2	174.0
IL-6 (pg/ml)	104.0	111.0	66.7	68.0
VAS	10	10	7	5
PGA	8	5	5	5
SF-36	49.31	61.81	43	46.25
Treatment				
IFX	5 mg/kg every 6–8 weeks for 6 months/5 mg/kg every 12 weeks	5 mg/kg every 8 weeks	5 mg/kg every 6–8 weeks for 6 months/5 mg/kg every 12 weeks	3 mg/kg every 8 weeks for 6 months/3 mg/kg every 12–16 weeks
MTX	15 mg weekly for 6 months/12.5 mg weekly	10 mg weekly	15 mg weekly for 6 months/discontinuation due to side effects	10 mg weekly for 6 months/discontinuation due to side effects
Prednisone	Not used	Not used	Not used	15 mg/day tapered to 5 mg/day

*WBC* white blood cells, *CRP* C-reactive protein, *ESR* erythrocyte sedimentation rate, *VAS* visual analogue scale, *PGA* physician global assessment, *SF-36* Short Form-36, *IFX* infliximab, *MTX* methotrexate

### Patient 1

**Fig. 1:**
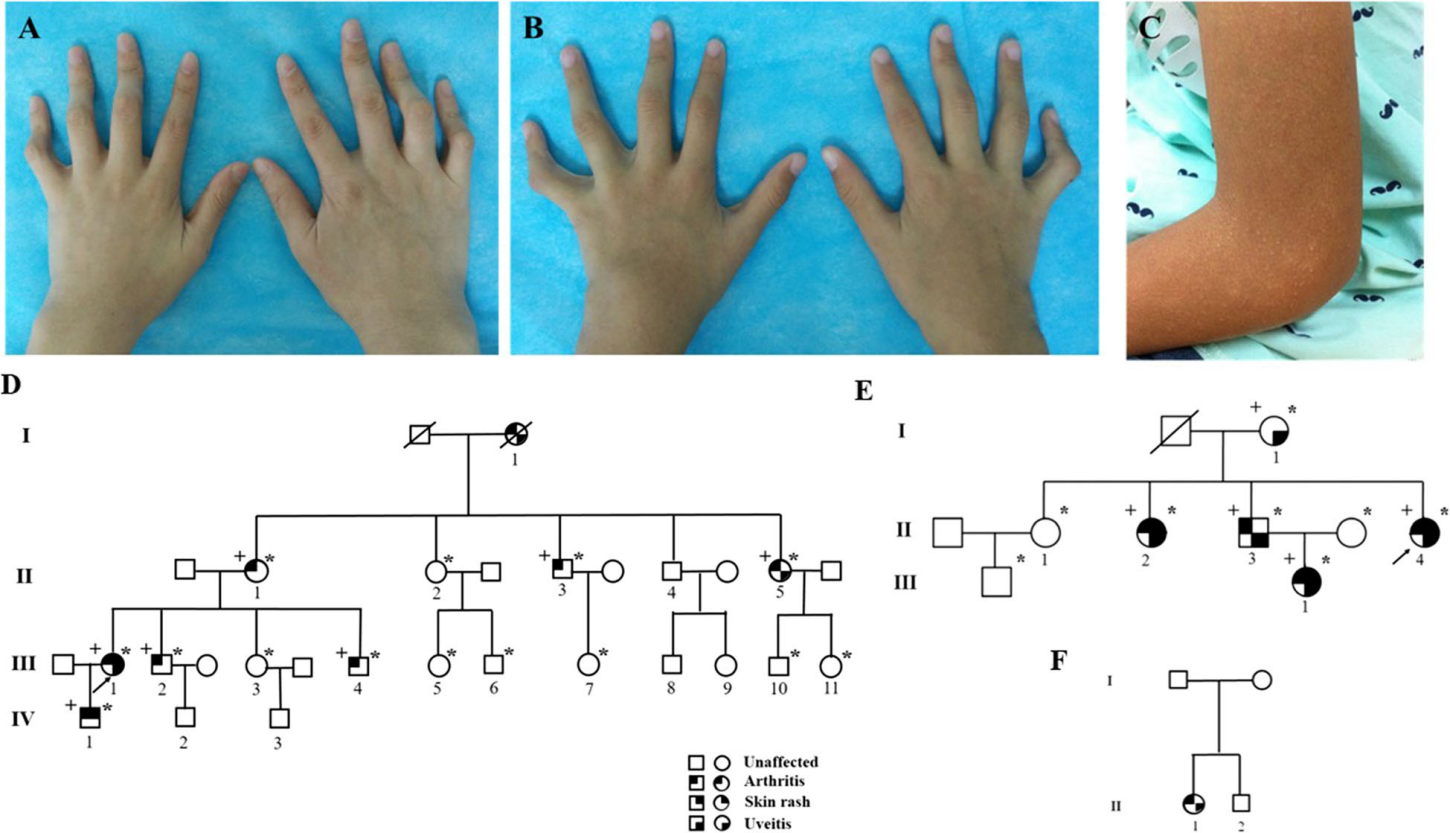
Pedigrees and phenotypes of Chinese patients with BS. Camptodactyly of patients 1 (**a**) and 2 (**b**); papules on the upper limbs of patient 2 (**c**); pedigrees of patients 1 and 2 (**d**), 3 (**e**), and 4 (**f**)

**Fig. 2:**
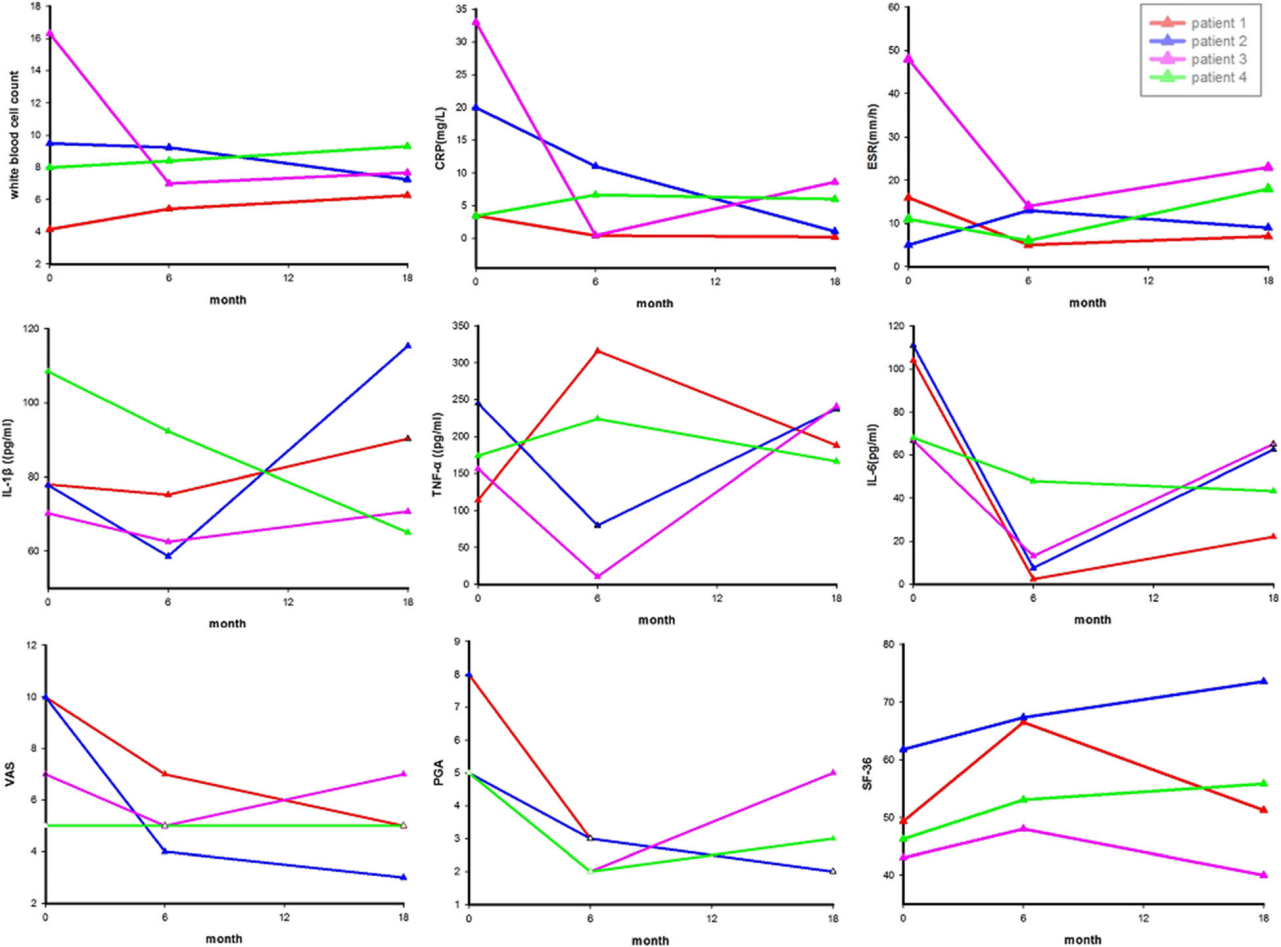
Changes in disease activity and inflammatory markers of the patients. Overall, WBC, CRP, ESR, IL-1β, IL-6, VAS, and PGA decreased, and SF-36 increased at the first follow-up of 6 months. TNFα decreased at the first follow-up in patients 2 and 3, but increased in patients 1 and 4. At the last visit of 18 months, WBC, ESR, and CRP remained normal in all except patient 3, whose ESR and CRP increased to above the normal range. Among all the patients, serum levels of IL-1β, TNFα, and IL-6 were higher than those at the first follow-up, except patient 4. For patients 1 and 2, VAS and PGA reduced further at the last follow-up, while for patients 3 and 4, they slightly increased. CRP C-reactive protein, ESR erythrocyte sedimentation rate, VAS visual analogue scale, PGA physician global assessment, SF-36 Short Form-36

A 32-year-old Chinese Han woman presented with dermatitis, arthritis, uveitis, and intermittent fever for 26 years. She had chronic polyarthritis involving bilateral joints of the hands, wrists, elbows, knees, and ankles since the age of 6, which resulted in camptodactyly (Fig. 1a). She also developed persistent bilateral panuveitis since the age of 12, which caused atrophy of both eyeballs and eventually complete loss of vision. She had papular rashes on extremities and intermittent fever. She had a family history of similar symptoms (Fig. 1d). A heterozygous R334W variant in the *NOD2* gene was identified and the diagnosis of BS was confirmed. Combination treatment of corticosteroids and disease-modifying antirheumatic drugs (DMARDs) such as methotrexate, leflunomide, and cyclosporine over 5 years had no effect. Laboratory evaluation of disease activity at diagnosis is shown in Table 1. She was treated with IFX (5 mg/kg) every 6 to 8 weeks at the beginning plus methotrexate 15 mg every week, with a satisfactory response for the polyarthritis and dermatitis. ESR and CRP rapidly decreased to normal levels after therapy. After 6 months, IFX was changed to 5 mg/kg every 12 weeks plus methotrexate 12.5 mg every week. At the last follow-up after IFX therapy of 18 months, her disease maintained stable (Fig. 2).

### Patient 2

This 8-year-old Chinese Han boy was the son of patient 1. He suffered from polyarthritis and papules on his four limbs at 6 months after birth and gradually developed camptodactyly (Fig. 1b, c) without uveitis or fever. He was treated with IFX (5 mg/kg) every 8 weeks plus methotrexate 10 mg every week, and the symptoms relieved rapidly (Fig. 2). At 18-month follow-up, he was symptom-free, and IFX was continued every 12–16 weeks.

### Patient 3

A 36-year-old Chinese Han woman complained of uveitis, dermatitis, and arthritis for nearly 30 years. She had chronic bilateral panuveitis since the age of 7, which led to blindness 7 years later. She suffered from widespread intermittent erythematous maculopapular rash since the disease onset. She also developed persistent polyarthritis since the age of 13, without obvious deformity. She had a positive family history of BS (Fig. 1e). Genetic testing identified a heterozygous *NOD2* R334W variant. The patient only received irregular NSAIDs for her arthritis with partial relief. ESR and CRP were elevated at baseline (Table 1). She received IFX (5 mg/kg) every 6 to 8 weeks plus methotrexate 15 mg every week. Polyarthritis and dermatitis subsided rapidly, and the acute phase reactants decreased at a 6-month follow-up. But she stopped methotrexate because of severe gastrointestinal side effects, and IFX was tapered to every 12 weeks due to financial limitations. At 18 months, her symptoms relapsed (Fig. 2) and IFX was given every 8 weeks again.

### Patient 4

A 25-year-old Chinese Han woman developed persistent polyarthritis since the age of 6, involving bilateral joints of the hands and knees, and gradually developed interphalangeal joint flexion. At the age of 20, bilateral uveitis occurred and her vision gradually decreased. She had no dermatitis or fever. She denied family history of BS (Fig. 1f). A de novo heterozygous R334Q variant was identified in the *NOD2* gene. Cyclosporine with prednisone had no effect for the polyarthritis and uveitis. IFX (3 mg/kg) every 8 weeks plus methotrexate 10 mg every week was given. Her symptoms were well controlled at a 6-month follow-up, and prednisone was tapered from 15 mg per day to 5 mg per day. Methotrexate was stopped due to gastrointestinal side effects, and IFX was reduced to every 12–16 weeks since then, but the disease relapsed at her last follow-up at 18 months (Fig. 2).

### Literature review

According to the 38 English-language publications, 62 patients with BS were reported who underwent TNFα inhibitor therapy. Among the 62 patients, IFX was used in 31 patients, adalimumab in 24, and etanercept in 7. IFX was well tolerated in 27 patients [11–22], while 2 patients still had uveitis [7, 23], and the other 2 experienced an adverse drug reaction to IFX infusion [21]. Adalimumab was useful in 21 BS patients [9, 14, 23–28], although the other 3 patients did not get well controlled [21, 29, 30]. Five BS patients who were given etanercept yielded good responses [9, 24, 31, 32], but 2 patients discontinued because of etanercept-induced myelopathy [33] or an exacerbation of arthritis [14].

## Discussion

Since it is difficult to differential BS from other rheumatic diseases such as rheumatoid arthritis, juvenile idiopathic arthritis, and Behçet’s disease, the diagnosis of BS is a great challenge to rheumatologists, especially in China. For instance, Behçet’s disease can manifest as arthritis, dermatitis, and uveitis as well, although other heterogenic features including oral and genital ulcers, arterial aneurysms, venous and arterial thrombosis, central nervous system involvement, and intestinal ulcers are prominent, and there is no *NOD2* gene mutation. Although there have been some reports of BS case series [6–8], including ours, the incidence of BS in the Chinese population is still unclear. In this study, we found that the duration of diagnosis delay was approximately 20 years. It suggests there may be underdiagnoses of BS in the Chinese population. On the other hand, our previous research showed that the clinical manifestations of BS in Chinese were milder and incomplete compared with those in Caucasians [8]. Meanwhile, one of the patients in our study was a sporadic case with a de novo *NOD2* variant. These findings suggest that BS should be considered and gene analysis should be promoted for patients with one or more manifestations among the clinical triad at disease onset, whether or not there is a family history.

Owing to the low incidence of BS, there have been no controlled clinical studies dealing with therapies for this disorder. Case series of BS concerning the treatments were mainly based on personal experience. High dose of glucocorticoids and immunosuppressants are effective in some patients, but the long-term use may cause severe side effects, especially to young patients. Moreover, immunosuppressants are ineffective in some BS patients and it may be difficult to reduce the dosage of steroids. As we know, NOD2 is an intracellular bacterial sensor protein of the NOD-like receptor (NLR) family which recognizes muramyl dipeptide (MDP), undergoes oligomerization, and interacts with receptor-interacting serine/threonine-protein kinase 2 (RIP2) [2]. Consequent phosphorylation of RIP2 results in the activation of NF-κB, thus inducing the production of pro-inflammatory cytokines, chemokines, and adhesion molecules to protect the host from infection and participate in the regulation of inflammation [34]. Meanwhile, it was reported that the levels of IL-1β, IL-6, and TNFα in plasma of BS patients were significantly higher than those of healthy control [35]. Thus, biologic therapy may be beneficial for BS. Recently, biologic agents such as IL-1 blockers, tocilizumab, and TNFα inhibitors have shown promising therapeutic response in refractory patients [4, 7, 9, 10, 29, 36]. Unfortunately, IL-1 blockers are unavailable in China, and the cost of tocilizumab is high. Therefore, TNFα inhibitors are the most commonly used biologic therapy in BS at present in China. IFX, which is a chimeric monoclonal antibody targeted against both receptor-bound and free TNFα, appears to be very effective in BS when combined with low-dose prednisone and low-dose methotrexate [18, 23]. In our literature review, IFX was also well tolerated. In this study, all of the four patients received IFX plus methotrexate, and all achieved clinical remission of skin lesions and polyarthritis rapidly, as well as normalization of ESR and CRP and improvements in VAS, PGA, and SF-36, at the first follow-up of 6 months. Thus, IFX showed a favorable effect on BS.

Despite the potential benefits of the biologics, future development of other potentially therapeutic agents is needed. Intriguingly, NOD2 functions through three signaling pathways, including mitogen-activated protein kinases (MAPK), NF-kB, and autophagy, after interacting with RIP2 and ATG16L1 [2]. From this point of view, the RIP2-specific inhibitors gefitinib and erlotinib which have been used in cancer therapy may be promising in the treatment of BS [37]. Nevertheless, a pilot study highlighted the ability of thalidomide, which may contribute to NF-*κ*B in BS pathogenesis, to improve symptoms in a few BS patients [9].

Since BS is a progressive disorder, diagnosis delay often leads to severe sequelae such as joint deformities and blindness. Hence, early treatment based on early diagnosis is important to avoid physical disability in BS patients. As in our study, patient 2 was diagnosed at an early stage, when he had only arthritis without uveitis and joint deformities. He obtained a complete clinical remission on TNFα inhibitor without the use of systemic corticosteroids. However, his mother, patient 1 in our study, despite a relatively satisfactory response to IFX, had missed the best opportunity for biological agents, and the joint deformities and blindness were irreversible. For BS patients, the main therapeutical goal is to avoid blindness and joint deformities and to improve quality of life as well. Indeed, patients 3 and 4 in our study had a good response to IFX, yet the disease relapsed after they lengthened the interval of medication and discontinued methotrexate. These results suggest that early intervention and regular medication is essential to improve patients’ quality of life and disease prognosis.

In conclusion, BS is often misdiagnosed prior to genetic analysis. It should be considered in patients with childhood-onset polyarthritis, uveitis, and dermatitis. Early recognition and effective treatment of BS are very important to avoid the irreversible organ damage (such as loss of vision and joint deformities). TNFα inhibitors such as IFX may be a promising approach in the management of BS patients who have unsatisfactory response to corticosteroids and traditional immunosuppressants. However, due to the sample limitation of our study, more clinical trials are required.

## Data Availability

All data generated or analyzed during this study are included in this published article.

## Abbreviations

BS: Blau syndrome
CRP: C-reactive protein
DMARDs: Disease-modifying antirheumatic drugs
ESR: Erythrocyte sedimentation rate
IFX: Infliximab
IL: Interleukin
NOD2: Nucleotide-binding oligomerization domain containing 2
NSAIDs: Non-steroid anti-inflammatory drugs
PGA: Physician global assessment
SF-36: Short Form-36
TNF: Tumor necrosis factor
VAS: Visual analogue scale
WBC: White blood cell count
